# A Putative Serine Protease is Required to Initiate the RIPK3-MLKL—Mediated Necroptotic Death Pathway in Neutrophils

**DOI:** 10.3389/fphar.2020.614928

**Published:** 2021-01-21

**Authors:** Xiaoliang Wang, Damjan Avsec, Aleš Obreza, Shida Yousefi, Irena Mlinarič-Raščan, Hans-Uwe Simon

**Affiliations:** ^1^ Institute of Pharmacology, University of Bern, Inselspital, INO-F, Bern, Switzerland; ^2^ Faculty of Pharmacy, University of Ljubljana, Ljubljana, Slovenia; ^3^ Department of Clinical Immunology and Allergology, Sechenov University, Moscow, Russia

**Keywords:** necroptosis, neutrophil, serine protease, signal transduction, small molecule inhibitor

## Abstract

Adhesion receptors, such as CD44, have been shown to activate receptor interacting protein kinase-3 (RIPK3)—mixed lineage kinase-like (MLKL) signaling, leading to a non-apoptotic cell death in human granulocyte/macrophage colony-stimulating factor (GM-CSF) – primed neutrophils. The signaling events of this necroptotic pathway, however, remain to be investigated. In the present study, we report the design, synthesis, and characterization of a series of novel serine protease inhibitors. Two of these inhibitors, compounds 1 and 3, were able to block CD44-triggered necroptosis in GM-CSF-primed neutrophils. Both inhibitors prevented the activation of MLKL, p38 mitogen-activated protein kinase (MAPK) and phosphatidylinositol 3’—kinase (PI3K), hence blocking the increased levels of reactive oxygen species (ROS) required for cell death. Although compounds one and three partially inhibited isolated human neutrophil elastase (HNE) activity, we obtained no pharmacological evidence that HNE is involved in the initiation of this death pathway within a cellular context. Interestingly, neither serine protease inhibitor had any effect on FAS receptor-mediated apoptosis. Taken together, these results suggest that a serine protease is involved in non-apoptotic CD44-triggered RIPK3-MLKL-dependent neutrophil cell death, but not FAS receptor-mediated caspase-dependent apoptosis. Thus, a pharmacological block on serine proteases might be beneficial for preventing exacerbation of disease in neutrophilic inflammatory responses.

## Introduction

Neutrophils are the most abundant leukocytes in human blood and play an essential role in innate immunity (Simon, 2003; Nauseef and Borregaard, 2014; Wang et al., 2018). Caspase-dependent apoptosis represents the most common form of both physiological and pathological cell death in neutrophils (Simon, 2003; Conus et al., 2008; Geering and Simon, 2011). In the resolution of inflammation, neutrophils rapidly undergo apoptosis and are removed by other phagocytic cells. Therefore, neutrophil apoptosis is considered to be an option for limiting tissue damage by preventing the release of histotoxic contents from dying cells (Simon, 2003; Geering and Simon, 2011). However, other types of neutrophil death have also been reported, for example necroptosis (Wang et al., 2016; Barbosa et al., 2018; Nakazawa et al., 2018; Wang et al., 2018; Zhou et al., 2018). Neutrophil necroptosis is a regulated form of necrosis, which is dependent on receptor interacting protein kinase-3 (RIPK3) and mixed lineage kinase-like (MLKL) activities (Wang et al., 2018). At the inflammatory sites, when neutrophils are activated by cytokines and other inflammatory mediators, as well as through adhesion receptors, neutrophil necroptosis can be triggered, most likely causing host tissue damage (Wang et al., 2018). Thus, necroptotic neutrophils are considered highly detrimental for the resolution of inflammation owing to the consequent release of toxic cellular contents and the potential escape of pathogens into the surroundings (Geering et al., 2013; Wang et al., 2018).

The mode of cell death has consequences for the surrounding environment and inflammatory responses. This is especially relevant for neutrophils, which contain granules filled with reactive chemicals and enzymes (Cowland and Borregaard, 2016; Benarafa and Simon, 2017). Clearly, a necrotic neutrophil may induce inflammatory responses by the immediate release of danger-associated molecular patterns (DAMPs), but also by causing tissue damage (Geering et al., 2013; Wang et al., 2018). Therefore, understanding the molecular mechanisms of non-apoptotic neutrophil death pathways may provide specific targets for potential therapeutic intervention in infectious, inflammatory and autoimmune diseases. Non-apoptotic neutrophil death pathways can be triggered in cytokine-primed neutrophils by ligation of surface receptors such as CD44 (Mihalache et al., 2011; Wang et al., 2016), CD11b (Wang et al., 2016), CD18 (Wang et al., 2016), CD15 (Wang et al., 2016), Siglec-9 (von Gunten et al., 2005), or CD89 (Wehrli et al., 2014).

Using a pharmacological approach, the activation of the RIPK3—MLKL signaling pathway has been shown to be essential for human neutrophils to undergo such a non-apoptotic cell death, suggesting that this type of death uses elements of the necroptotic pathway (Wang et al., 2016).

Serine proteases constitute almost one third of all proteases (Hedstrom, 2002), and probably they are the enzymes that have been most studied in details (Page and Di Cera, 2008; Soualmia and El Amri, 2018). Serine proteases are characterized by a catalytic serine residue at the active site and have been shown to play essential roles in biological processes in blood coagulation, development, cell death, inflammation, immune response, and signal transduction (Heutinck et al., 2010; Turk et al., 2012; Benarafa and Simon, 2017; Soualmia and El Amri, 2018). In the present report, we have investigated the effect of novel, newly synthesized serine protease inhibitors on neutrophil death *in vitro*. Two inhibitors, designated here as compounds 1 and 3, were shown to be able to inhibit CD44-induced necroptotic cell death in granulocyte/macrophage colony-stimulating factor (GM-CSF)-primed human neutrophils. In contrast, FAS receptor-mediated neutrophil apoptosis was not blocked. Interestingly, both compounds 1 and 3 inhibited MLKL, p38, PI3K and NADPH oxidase activation. Moreover, similar to compounds 1 and 3, N-*p*-tosyl-l-phenylalanine chloromethyl ketone (TPCK) blocked the same signaling events leading to neutrophil death. Taken together, these results suggest that serine protease activity is required to activate MLKL in neutrophils, leading to high ROS production and subsequent non-apoptotic cell death.

## Materials and Methods

### Design and Synthesis of the Compounds

A group of structurally related hydrazide or sulfonohydrazide derivatives was chosen for current study. The design, synthesis and spectroscopic data of compounds 2, 5–14 (structures presented in Supplementary Table S1) have been previously described (Obreza et al., 2004a; Obreza et al., 2004b; Smolnikar et al., 2007). All chemicals were obtained from commercial sources (Carlo Erba, Acros Organics, Emsure, TCI, AK Scientific, Sigma Aldrich, Merck and Fluka) and were used without further purification. Analytical thin-layer chromatography (TLC) was performed on Merck silica gel (60F_254_) plates (0.25 mm). Column chromatography was performed on silica gel 60 (Merck, particle size 0.040–0.063 mm). Melting points were determined on a Reichert hot stage microscope and are uncorrected. ^1^H– and ^13^C–NMR spectra were recorded on a Bruker AVANCE DPX_400_ spectrometer in CDCl_3_ or DMSO–*d*_6_ solution with TMS as internal standard. Chemical shifts are reported in ppm (*δ*) downfield from TMS. All the coupling constants (*J*) are in hertz. IR spectra were recorded on a PerkinElmer Spectrum BX System FT–IR spectrometer. Mass spectra were measured with Advion expression CMLS mass spectrometer with ESI ionization. High-performance mass spectra were obtained with a Exactive™ Plus Orbitrap Mass Spectrometer with ESI ionization. Reported yields are of purified products.

Compounds 1 were synthesized according to the modified procedure in which 4-aminopiperidine was used instead of azetidine (Obreza et al., 2004a). Compound 3 was prepared in a three step synthesis from corresponding aldehyde and hydrazide. Cyanobenzaldehyde (1.31 g; 10.0 mmol) and 2-naphthohydrazide (1.86 g; 10.0 mmol) were dispersed in 20 ml of anhydrous ethanol and refluxed for 5 h. Approximately 50% of the solvent was then removed *in vacuo* and the residue diluted with water. The resulting white solid was filtered and dried.

N′-(4-cyanobenzylidene)-2-naphthohydrazide (2.06 g; 6.89 mmol) was dissolved in 25 ml of methanol. 200 mg of 10% palladium on activated charcoal was added to the solution with constant influx of argon. The reaction mixture was hydrogenated overnight at 2 bar for 3 h at room temperature. After the catalyst was removed, the solvent was evaporated under reduced pressure.

N′-(4-cyanobenzyl)-2-naphthohydrazide (1.50 g; 4.98 mmol) was dissolved in anhydrous ethanol. Hydroxylamine hydrochloride (402 mg; 5.78 mmol) and K_2_CO_3_ (1.30 g; 9.42 mmol) were added and the mixture was stirred for 6 h at 59 °C. The solvent was removed in vacuo and the residue dissolved in mixture of ethyl acetate (30 ml) and water (20 ml). Phases were separated and organic phase washed with 10% citric acid (20 ml), concentrated NaHCO_3_ (20 ml) and brine (20 ml), dried over sodium sulfate and the solvent removed in vacuo. The crude product was re-crystallized from ethanol.

The characterization of the compounds by spectroscopy and enzyme kinetics are described in the Supplementary Material.

### Reagents and Antibodies

Anti-CD44 mAb (clone A3D8), GW311616A, dihydrorhodamine 123 (DHR), the cell permeable inhibitor of human leukocyte elastase, N-(methoxysuccinyl)-alanyl-alanyl-prolyl-valyl-chloromethyl ketone (AAPVCK), and the elastase inhibitor IV were from Sigma-Aldrich (Buchs, Switzerland). The F(ab’)_2_ fragments of the secondary goat anti-mouse (GaM) Ab was purchased from Jackson ImmunoResearch Laboratories (Milan Analytica; Roche Diagnostics, Rotkreuz, Switzerland). GM-CSF was supplied by Novartis Pharma (Nürnberg, Germany). Wortmannin and diphenylene iodonium (DPI) chloride were purchased from Calbiochem Novabiochem (La Jolla, CA, United States). Q-VD-Oph was from SM Biochemicals (Anaheim, California, United States). PD169316 was obtained from Merck Millipore (Darmstadt, Deutschland). Anti-FAS agonistic mAb (CH11) was purchased from MBL International (Woburn, MA, United States). GSK′872 was from Glixx Laboratories (Southborough, MA) and GW806742X was from Adipogen AG (Liestal, Switzerland). Rabbit anti-MLKL and rabbit anti-phospho-MLKL (phospho Ser358) mAb were from Abcam (Cambridge, United Kingdom). Rabbit anti-caspase-3, rabbit anti-AKT, rabbit anti-phospho-Ser473-AKT, rabbit anti-p38, and rabbit anti–phospho-Thr180/Tyr182-p38 were all from Cell Signaling Technology (Danvers, MA, United States). Anti-GAPDH mAb was obtained from Chemicon International (Chandlers Ford, United Kingdom). HRP-coupled secondary Abs (anti-mouse IgG and anti-rabbit IgG) were from GE Healthcare (VWR, Switzerland). Luminata™ Forte Western HRP substrate was purchased from Millipore Corporation (Billerica, MA, United States). The monovalent Smac mimetic compound AT-406 was from Selleckchem (Houston, TX, United States). Human TNF-α was purchased from R and D Systems (Abingdon, United Kingdom). Abz-APEEIMRRQ-EDDnp was from PeptaNova GmbH (Sandhausen, Germany). White polypropylene-well, 96-well microplates (Hard-Shell Thin-Wall Microplates, cat. no. Hardshell microplaques 96 black shell, white wells, # 80050379) were from Bio-Rad Laboratories (Hercules, CA94547, United States).

### Cells

Peripheral blood neutrophils were purified from healthy normal individuals by Ficoll-Hypaque centrifugation, as described previously (Mihalache et al., 2011; von Gunten et al., 2005; Wang et al., 2016; Stojkov et al., 2017). The purity of the isolated human neutrophil populations was always >95%, as assessed by staining with Diff-Quik (Baxter, Düdingen, Switzerland) and light microscopic analysis. Caspase-8-deficient Jurkat cells were a kind gift of Dr T. Brunner from University of Konstanz, Germany.

### Cell Cultures

Human blood neutrophils were cultured at 1 × 10^6^/ml in RPMI 1640 medium plus GlutaMAX (Invitrogen) supplemented with 5% FCS and antibiotics in the presence and absence of GM-CSF (10 ng/ml), anti-FAS (1 μg/ml), TNF-α (50 ng/ml), Q-VD-Oph (20 μM), PD169316 (10 μM), wortmannin (100 nM), DPI (1 μM), GSK′872 (10 μM), GW806742X (5 μM), TPCK (10 μM), GW311616A (1, 10, and 20 μM), AAPVCK (10 μM), elastase inhibitor IV (10 μM) or the compounds 1–14 (the novel, synthesized serine protease inhibitors at the indicated concentrations). Pre-incubation with inhibitors and GM-CSF stimulation before ligation of adhesion molecules were performed for 30 min. Anti-CD44 (6 μg/ml) was added for 15 min prior to addition of GaM (20 μg/ml) for receptor ligation. Cells were cultured for the indicated time periods.

Caspase-8-deficient Jurkat cells were cultured at 1 × 10^6^/ml in RPMI 1640 medium plus GlutaMAX (Invitrogen) supplemented with 5% FCS and antibiotics in the presence and absence of compound 1 (40 μM), compound 3 (20 μM), TNF-α (20 ng/ml), Q-VD-Oph (20 μM), or the Smac mimetic AT-406 (100 nM). Pre-incubation with compound 1 or compound 3 before stimulation were performed for 30 min.

### Determination of Cell Death

Cell death was assessed by uptake of 25 μΜ ethidium bromide and flow cytometric analysis (FACSCalibur; BD Biosciences) (Mihalache et al., 2011; Geering et al., 2011; Wang et al., 2016; Amini et al., 2018). To determine whether cell death was apoptosis, redistribution of phosphatidylserine (PS) in the presence of propidium iodide (PI) was measured by flow cytometry (Mihalache et al., 2011; Geering et al., 2011).

### Oxidative Burst Measurements

Neutrophils were cultured as indicated and subsequently incubated with 1 μM DHR at 37 °C for 30 min, placed on ice, and analyzed by flow cytometry (Mihalache et al., 2011; Geering et al., 2011).

### Immunoblotting

Immunoblotting experiments were performed as previously described (Wang et al., 2016; Mihalache et al., 2011; Geering and Simon, 2011). Briefly, human neutrophils were lyzed in Triton lysis buffer (1% Triton X-100, 150 mM NaCl, 50 mM Tris-HCl [pH 7.4], and 1 mM EDTA) containing both protease (protease inhibitor cocktail P8340 and PMSF; Sigma-Aldrich) and phosphatase inhibitors (1 μM okadaic acid, 1 mM Na_3_VO_4_, and 5 mM NaF). The lysates were heated at 95 °C for 5 min. Proteins were separated by SDS-PAGE and electroblotted onto polyvinylidene difluoride membranes (Immobilion-P; Millipore, Bedford, MA). The membranes were routinely blocked in TBS containing 0.1% Tween-20 and 5% nonfat dry milk, followed by incubation overnight with the indicated Abs at 4 °C. The next morning, samples were further incubated with the appropriate HRP-conjugated secondary Ab for 1 h at room temperature. Filters were developed using an ECL technique (ECL-Kit; GE Healthcare) according to the manufacturer’s instructions.

### Human Neutrophil Elastase Activity Assay

Measurement of HNE activity on the supernatant of resting or activated human neutrophils was performed by using a Abz-APEEIMRRQ-EDDnp fluorescence resonance energy transfer (FRET) substrate (Korkmaz et al., 2008). 150 μL of neutrophils (2 × 10^5^ cells) were cultured as indicated in RPMI 1640 medium and subsequently centrifuged at 2,000 RPM for 4 min at room temperature. The supernatants were then collected and transtered in a white polypropylene-well, 96-well microplate. Reactions were started by adding Abz-APEEIMRRQ-EDDnp to a final concentration of 20 µM in a final reaction volume of 150 µL. Fluorescent activity was immediately measured at 320 nm excitation/420 nm emission using a spectrofluorometer (SpectraMax M2; Molecular Devices, Biberach an der Riß, Germany) as described previously (Korkmaz et al., 2008). Relative fluorescence was obtained after subtraction of the corresponding fluorescence of medium without cells at the corresponding time point and subsequent norming of the values to initially measured fluorescence.

### Statistics

Data are presented as means ± SEM. One-way ANOVA followed by a Tukey multiple comparison test was applied. A *p* value < 0.05 was considered statistically significant.

## Results

### Chemical Characterization of a Series of Novel Serine Protease Inhibitors

The general structure of all 14 compounds (Supplementary Table S1) consists of the highly hydrophobic and relatively rigid 2-naphthohydrazide or naphthalene-2-sulfonohydrazide moiety, with meta or para substituted benzyl or piperidinomethyl group on N′. The main difference between the compounds is in the functional group on the phenyl or piperidine ring that defines the acido-basic properties of compounds. In the cytoprotective compounds 1 and 3, a slightly basic amidoxime is present on para position of a benzyl group. Compound 1 has an additional six membered ring attached through carbonyl group, making it larger in size compared to compound 3 (Supplementary Table S1).

### Differential Effects of Serine Protease Inhibitors on Spontaneous Neutrophil Death *in vitro*

There is growing evidence for a role for serine proteases in the regulation of neutrophil death (Conus and Simon, 2008; Loison et al., 2014a; Loison et al., 2014b; Baumann et al., 2013; Benarafa and Simon, 2017). To obtain further insight into this involvement, we tested the effect of 14 potential serine protease inhibitors on neutrophil death. Neutrophils, isolated from peripheral blood of healthy donors, are known to undergo apoptosis *in vitro*, a process called spontaneous or constitutive apoptosis (Simon, 2003; Benarafa and Simon, 2017). Using this *in vitro* system, we treated neutrophils with the compounds in a concentration-dependent manner and measured viability after 24 h (Supplementary Figure S1). As a control, GM-CSF was used to delay spontaneous neutrophil apoptosis (Dibbert et al., 1999; Saba et al., 2002; Simon, 2003). Anti-FAS antibody and TNF-α, both known as triggers of neutrophil apoptosis (Geering and Simon, 2011; Geering et al., 2011), were applied as additional controls. Approximately 40% of the untreated neutrophils underwent cell death, which is referred as spontaneous neutrophil apoptosis. Compounds 1 and 3 inhibited spontaneous neutrophil death with similar efficacy as GM-CSF and increased the average neutrophil survival at optimal concentrations by approximately 20% (Supplementary Figure S1). To determine the optimal inhibitory concentration, we performed concentration-dependent experiments using compound 1 at concentrations ranging from 1 to 75 µM and compound 3 ranging from 1 to 30 µM. These experiments confirmed the anti-death activity of both compounds. Optimal inhibition of neutrophil death was achieved with 40 µM compound 1 and 20 µM compound 3 (Supplementary Figure S2). Taken together, these data suggest that compounds 1 and 3 target serine proteases involved in spontaneous apoptosis. Therefore, we subsequently investigated the effects of both compounds on different neutrophil death pathways.

### Compounds 1 and 3 Block Spontaneous Neutrophil Apoptosis, but Not FAS-Mediated Apoptosis

Phosphatidylserine (PS) is normally confined to the inner plasma membrane leaflet. However, PS appears on the external leaflet in apoptotic cells. Although it has been shown that PS can be externalized on the surface of cells undergoing other forms of cell death including necroptosis (Gong et al., 2017; Oberst et al., 2017; D’Cruz et al., 2018; Speir et al., 2020), the redistribution of PS is still considered to be a feature of apoptotic cells and can be detected by Annexin V binding to exposed PS. Moreover, it has been demonstrated that the type of both spontaneous and FAS-triggered neutrophil death is apoptosis (Simon, 2003). To investigate whether compounds 1 and 3 decrease spontaneous neutrophil death by inhibition of apoptosis, neutrophils were analyzed by Annexin V/propidium iodide (PI) assay. Compounds 1 and 3 decreased the percentage of apoptotic neutrophils (Annexin V^+^/PI^−^) in untreated neutrophils (Supplementary Figure S3A). In contrast, FAS-induced apoptosis was not blocked by either compounds 1 or 3 (Supplementary Figure S3A). In contrast, the pan-caspase inhibitor Q-VD-Oph prevented FAS-induced death (Supplementary Figure S3B).

Caspase-3 is an effector caspase and its activation is crucial for FAS-mediated neutrophil apoptosis (Simon, 2003). In agreement with previously published work, we observed caspase-3 cleavage after 8 h treatment with anti-FAS antibody, suggesting caspase-3 activation as a consequence of FAS ligation (Supplementary Figure S3C) (Geering et al., 2011; Mihalache et al., 2011; Wang et al., 2016). While Q-VD-Oph prevented caspase-3 cleavage, neither compounds 1 and 3 had any effect (Supplementary Figure S3C, *left panel*). Compounds 1 and 3 had also no effect on cleaved caspase-3 in the absence of FAS activation (Supplementary Figure S3C, *right panel*). Taken together, though both compounds were able to block spontaneous apoptosis, we obtained no evidence for an involvement of serine proteases in FAS receptor-mediated apoptosis in our system.

### CD44-Induced ROS Production and Subsequent Necroptosis in GM-CSF-Primed Neutrophils Require Serine Protease Activity

It has been previously shown that multiple triggers can induce a non-apoptotic death in cytokine-primed neutrophils which uses components of a death pathway called necroptosis (von Gunten et al., 2005; Mihalache et al., 2011; Wehrli et al., 2014; Wang et al., 2016; Wang et al., 2018). In neutrophils, this pathway results in the activation of the NADPH oxidase, resulting in the production of high levels of ROS. Pharmacological or genetic inactivation of the NADPH oxidase prevents this non-apoptotic neutrophil death (von Gunten et al., 2005; Mihalache et al., 2011; Wehrli et al., 2014; Wang et al., 2016; Wang et al., 2018). In this study, we used the death triggered by CD44 ligation, a system which has been well characterized previously (Mihalache et al., 2011; Wang et al., 2016).

**FIGURE 1:**
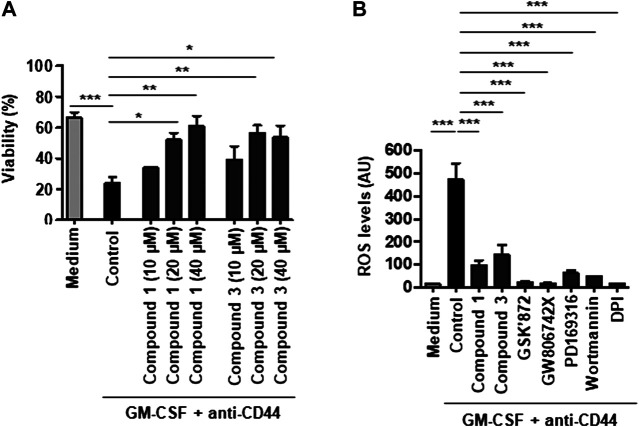
CD44-induced ROS production and subsequent necroptosis in GM-CSF-primed neutrophils require serine protease activity. **(A)** Concentration-dependent effect of serine protease inhibitors, compounds 1 or 3, on CD44-triggered necroptosis in GM-CSF-primed neutrophils. GM-CSF-primed neutrophils were precultured in the presence and absence of compound 1 (10, 20, or 40 μM) or compound 3 (10, 20, or 40 μM), and subsequently stimulated with anti-CD44 mAb for 24 h. Cell death was assessed by uptake of 25 μM ethidium bromide with flow cytometric analysis. **(B)** DHR oxidation assay. GM-CSF-primed neutrophils were precultured in the presence and absence of the serine protease inhibitor compounds 1 (40 μM) or 3 (20 μM), the RIPK3 inhibitors GSK′872 (10 μM), the MLKL inhibitors GW806742X (5 μM), the p38 inhibitor PD169316 (10 μM), the PI3K inhibitor wortmannin (100 nM), or the NADPH oxidase inhibitor DPI (1 μM) and subsequently stimulated with anti-CD44 mAb for 15 min before measured by flow cytometry. All values are means ± SEM of at least 3 independent experiments. *p < 0.05, **p < 0.01, ***p < 0.001.

In agreement with the previously published work (Mihalache et al., 2011; Wang et al., 2016), CD44-mediated cell death in GM-CSF-primed neutrophils (Figure 1A). Both compounds 1 and 3 inhibited CD44-triggered neutrophil necroptosis in a concentration-dependent manner. Optimal inhibition of neutrophil necroptosis was achieved with 40 µM compound 1 and 20 µM compound 3 (Figure 1A). We next analyzed ROS production following CD44 ligation of GM-CSF-primed neutrophils. Pharmacological inhibition of RIPK3 (GSK′872), MLKL (GW806742X), p38 MAPK (PD169316), PI3K (wortmannin), or NADPH oxidase (DPI) blocked ROS production as described previously (Wang et al., 2016). Both compounds 1 (40 μM) and 3 (20 μM) also strongly reduced ROS production induced in this death pathway (Figure 1B), suggesting that serine protease(s) are involved proximal to NADPH oxidase activation.

### Serine Protease Activity Is Required for MLKL Phosphorylation as Well as Subsequent p38 MAPK and PI3K Activation for CD44-Triggered Neutrophil Necroptosis

To gain insight into the signaling events leading to the block in ROS formation, we next addressed whether serine protease inhibition by compounds 1 and 3 modulates the phosphorylation of MLKL, p38 MAPK and AKT, signaling events known to be proximal to ROS production in CD44-triggered neutrophil necroptosis (Wang et al., 2016).

**FIGURE 2:**
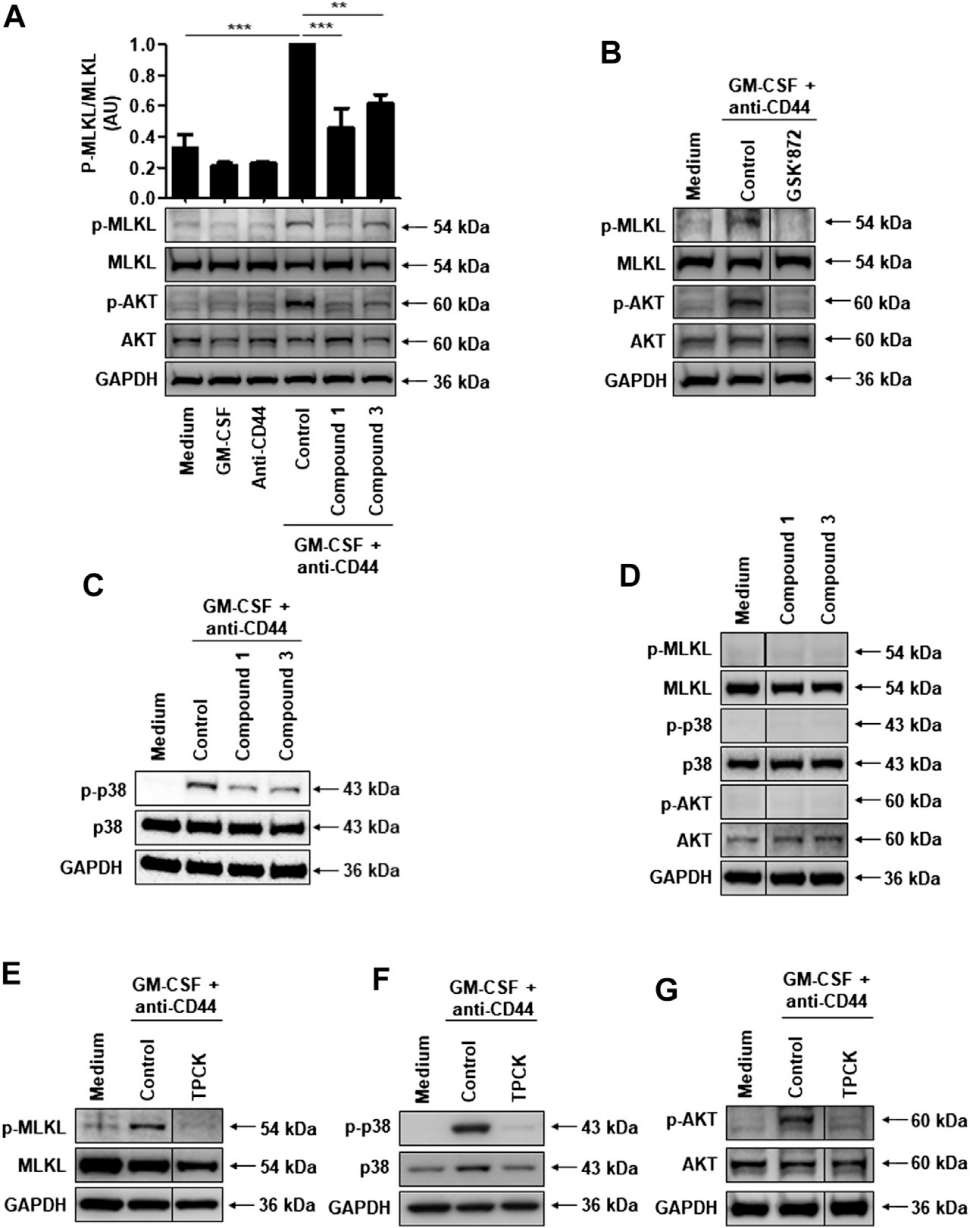
Serine protease activity is required for RIPK3-dependent MLKL phosphorylation, and subsequent p38 MAPK and PI3K activation in CD44-induced necroptosis in GM-CSF-primed neutrophils. **(A–C** and **E–G)** Immunoblotting. Neutrophils were cultured as indicated and subsequently stimulated with anti-CD44 mAb for 5 min. **(D)** Immunoblotting. Neutrophils were cultured in the presence and absence of compound 1 (40 μM) or compound 3 (20 μM) for 1 h. Cell lysates were analyzed by immunoblotting for phosphorylated MLKL, phosphorylated AKT, and phosphorylated p38 MAPK. MLKL, AKT, p38, and GAPDH expression levels were analyzed as loading controls. Representative immunoblots are shown (n ≥ 3). **(A)** *Upper panel*: *p*-MLKL protein expression levels were quantified relative to the control condition of MLKL. Data are from 3 independent experiments. **p < 0.01, ***p < 0.001.

CD44 ligation in GM-CSF-primed neutrophils induced the phosphorylation of MLKL as previously described (Wang et al., 2016), a signaling event which is inhibited by compound 1 or compound 3 (Figure 2A). However, the inhibition effect of compound 3 on MLKL seems less efficient compared to compound 1 (Figure 2A). To further investigate whether these two compounds can indeed significantly inhibit MLKL activity, we also analyzed the *p*-MLKL expression levels quantified to the control condition of MLKL. The results showed that both compounds 1 and 3 significantly inhibit MLKL activity in CD44-induced cell death system (Figure 2A). GSK′872, an inhibitor of RIPK3, is able to abrogate MLKL phosphorylation (Figure 2B), confirming that MLKL is indeed phosphorylated in a RIPK3-dependent manner in this system. Furthermore, we found that both compounds 1 and 3 can inhibit p38 MAPK and AKT phosphorylation (Figure 2A and C). Moreover, the inhibitors alone had no effect on the expression and phosphorylation of MLKL, p38 MAPK or AKT (Figure 2D). In addition, neither compound interacted directly with the RIPK3-MLKL complex, since they were not able to block necroptosis induced by combinatorial treatment with TNF-α, the Smac mimetic AT-406, and Q-VD-Oph in caspase-8-deficient Jurkat cells (Supplementary Figure S4) (Laukens et al., 2011; Amini et al., 2016).

These data imply that serine protease activity is involved in signaling events proximal to NADPH oxidase activation. We used TPCK, a broad-spectrum pharmacological serine protease inhibitor, to block MLKL, p38 MAPK and AKT activities. Indeed, TPCK completely blocked their phosphorylation (Figure 2E–2G). TPCK also blocked CD44-induced ROS production and subsequent necroptosis in GM-CSF-primed neutrophils (Supplementary Figure S5). Taken together, a serine protease activity is required for ROS production by activating MLKL, p38 MAPK or AKT phosphorylation events following CD44 ligation in GM-CSF-primed neutrophils. However, the responsible serine protease(s) remain(s) to be identified.

### Possible Serine Proteases Involved in CD44-Induced ROS and Cell Death

**FIGURE 3:**
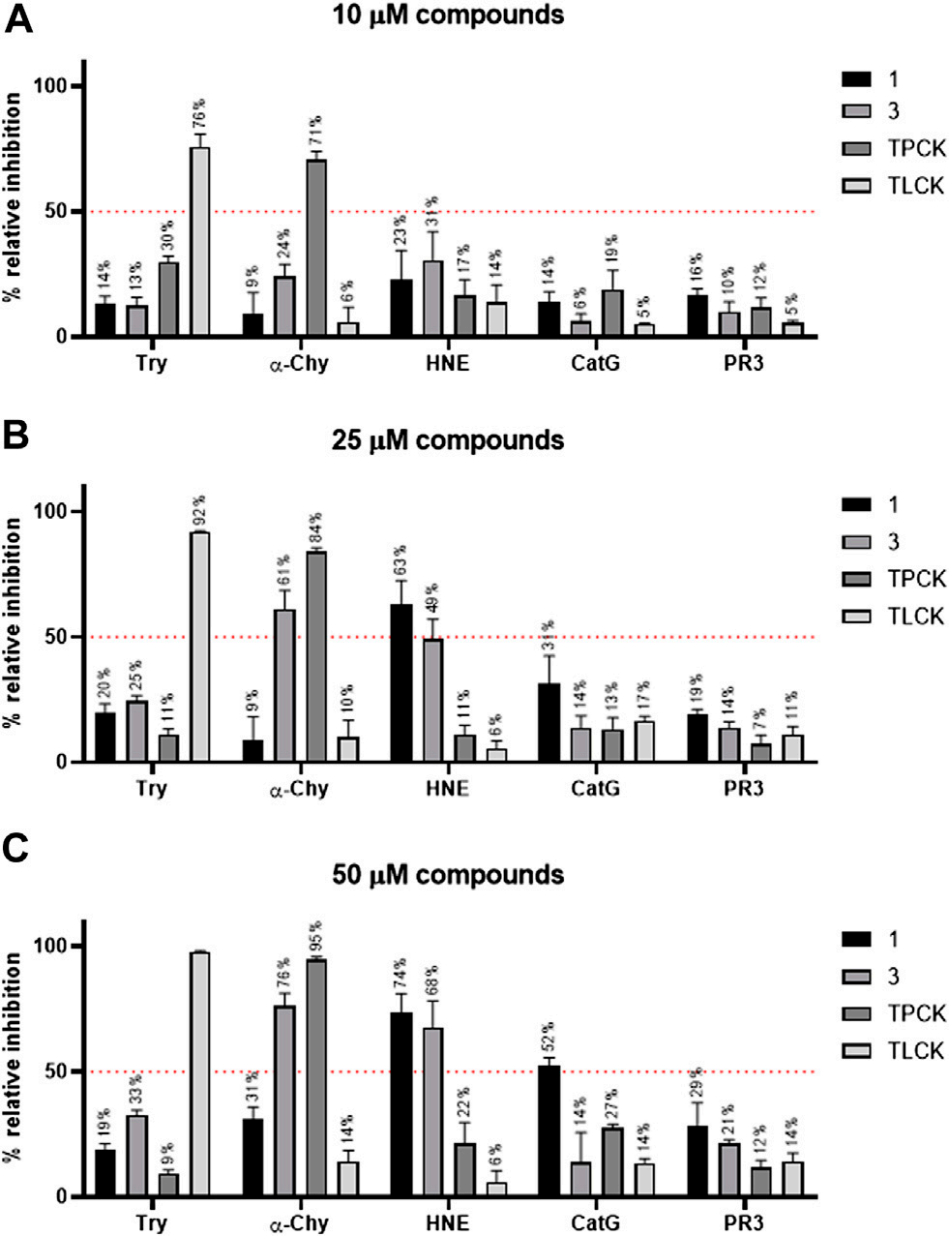
Comparative activity of compounds against five different serine proteases. Compound 1, 3, TPCK, and TLCK were incubated with trypsin (Try), α-chymotrypsin (α-Chy), human neutrophil elastase (HNE), cathepsin G (CatG), and proteinase 3 (PR3) at 37 °C for 30 min. Subsequently, reactions were started by adding an enzyme-specific substrate. Substrate hydrolysis was monitored in a kinetic mode for 60 min. The initial rates of reactions were used to calculate the % relative inhibition of the enzymes. Results are presented as mean ± SEM of 3 independent experiments, run in duplicate. **(A)** Relative inhibition (%) of serine proteases for 10 µM compound 1, 3, TPCK, and TLCK. **(B)** Relative inhibition (%) of serine proteases for 25 µM compound 1, 3, TPCK, and TLCK. **(C)** Relative inhibition (%) of serine proteases for 50 µM compound 1, 3, TPCK, and TLCK.

We next tested if compounds 1 and 3 were able to inhibit the serine proteases human neutrophil elastase (HNE), cathepsin G (CatG), and proteinase 3 (PR3) that are known to be present in azurophilic granules of neutrophils (Benarafa and Simon, 2017). The enzymes trypsin and α-chymotrypsin as well the inhibitors Nα-Tosyl-l-lysine chloromethyl ketone hydrochloride (TLCK) and TPCK served as controls. Compound 1, at 25 or 50 μM concentration, was able to inhibit 63% or 74% of HNE activity, respectively (Figure 3). At 50 μM concentration, compound 1 can inhibit 52% of CatG activity (Figure 3). In contrast, compound 1 was unable to inhibit PR3, trypsin or α-chymotrypsin activities (Figure 3). Compound 3, at 25 or 50 μM concentration, was able to inhibit 49% or 68% of HNE activity, respectively (Figure 3). In contrast, compound 3 did not inhibit CatG, PR3, and trypsin activities (Figure 3). However, at 25 and 50 μM, compound 3 was able to inhibit α-chymotrypsin activity (Figure 3). Looking at the structure-activity relationship, the main difference between compound 1 and 3 is in the additional piperidine ring present in compound 1 but not 3. This suggests that the selectivity toward CatG and α-chymotrypsin is a result of the presence or absence of the piperidine ring. On the other hand, both compound 1 and 3 inhibited at high concentrations partially the enzymatic activity of HNE, pointing to a possible proximal role of HNE in the RIPK3-MLKL – mediated necroptotic death pathway in neutrophils.

**FIGURE 4:**
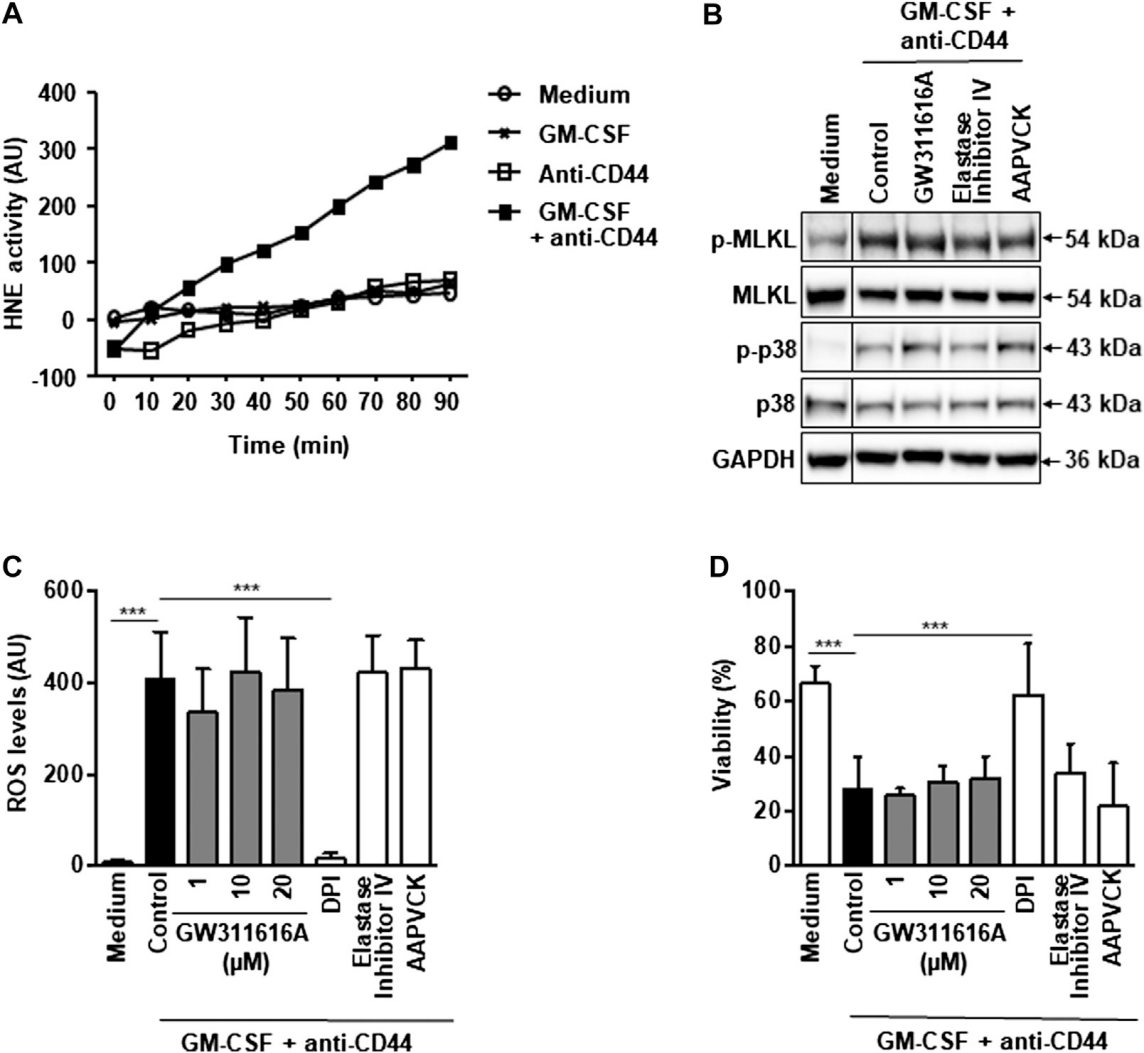
Pharmacological inactivation of HNE does not block CD44 death signaling in GM-CSF-primed neutrophils. **(A)** Enzymatic assay. Supernatant HNE activity can be measured upon CD44 activation in GM-CSF-primed neutrophils. Data are representative of 3 independent experiments. **(B)** Immunoblotting. Neutrophils were cultured as indicated and subsequently stimulated with anti-CD44 mAb for 5 min. Preincubation with GW311616A (10 μM), AAPVCK (10 μM), or elastase inhibitor IV (10 μM) was performed for 30 min. Cell lysates were analyzed by immunoblotting for phosphorylated MLKL and phosphorylated p38 MAPK. MLKL, p38 and GAPDH expression levels were analyzed as loading controls. **(C)** DHR oxidation assay. GM-CSF-primed neutrophils were precultured in the presence and absence of GW311616A (1, 10, and 20 μM), AAPVCK (10 μM), or elastase inhibitor IV (10 μM), and subsequently stimulated with anti-CD44 mAb for 15 min before measured by flow cytometry. DPI (1 μM) served as a control. **(D)** Viability assay. GM-CSF-primed neutrophils were precultured in the presence and absence of GW311616A (1, 10, and 20 μM), AAPVCK (10 μM), or elastase inhibitor IV (10 μM) for 30 min and subsequently stimulated with anti-CD44 mAb for 24 h. Cell death was assessed by uptake of 25 μM ethidium bromide with flow cytometric analysis. All values are means ± SEM of at least 3 independent experiments. ***p < 0.001.

We next investigated the HNE activity in the supernatants of GM-CSF-primed and CD44-activated neutrophils. Our results provided evidence that HNE is activated in the process of neutrophil activation, but an increased enzymatic activity did not occur before 30 min (Figure 4A). On the other hand, maximal ROS levels are seen at 15 min in GM-CSF-primed and CD44-activated neutrophils (Mihalache et al., 2011), suggesting that increased HNE activity is rather a late event. To further exclude that HNE activity is required to activate the RIPK3-MLKL – mediated necroptotic death in neutrophils, we tested if GW311616A and two additional well known HNE inhibitors are able to block the pathway. All three HNE inhibitors did not prevent the phosphorylation of MLKL and p38 (Figure 4B), ROS production (Figure 4C) and cell death (Figure 4D) in GM-CSF-primed and CD44-activated neutrophils. Therefore, it is unlikely that HNE is the proximal serine protease required to activate the RIPK3-MLKL pathway following death triggering. It should be noted that fibroblast activation protein-α (FAP-α), another serine protease expressed in neutrophils, acts in parallel to the RIPK3-MLKL pathway and is not able to activate it in the process of CD44-mediated neutrophil death (Wang et al., 2020).

## Discussion

Two novel serine protease inhibitors, compounds 1 and 3, can block CD44-triggered necroptosis in GM-CSF-primed human neutrophils by blocking RIPK3-MLKL, p38 MAPK, PI3K, and NADPH oxidase activation. In contrast to classical necroptosis (Chen et al., 2014; Dondelinger et al., 2014; Quarato et al., 2016; Zhao et al., 2016; Petrie et al., 2017), it seems that MLKL is here not involved in a pore-forming mechanism within the plasma membrane (Wang et al., 2016). In this cell death pathway present in human neutrophils, it rather acts within a signaling cascade leading to p38 mitogen-activated protein kinase (MAPK) and phosphatidylinositol 3′-kinase (PI3K) activation, followed by the production of high levels of reactive oxygen species (ROS), most likely owing to nicotinamide adenine dinucleotide phosphate (NADPH) oxidase activation (Wang et al., 2016). It should be noted, however, that in mouse neutrophils, classical necroptosis can be triggered by the SMAC-mimetic birinapant in combination with the pan-caspase inhibitor z-VAD-fmk whereby MLKL accumulates in the cell membrane (D’Cruz et al., 2018).

Our findings imply that a serine protease activity is required proximal of RIPK3-MLKL, p38 MAPK, PI3K, and NADPH oxidase activation. On the other hand, RIPK3 has been shown to activate the NLRP3 inflammasome, leading to IL-1β inflammatory responses (Lawlor et al., 2015; Weinlich et al., 2017). Therefore, there is a possibility that a RIPK3-NLRP3 inflammasome-dependent pathway is activated in human neutrophils following GM-CSF priming and adhesion receptor activation. Whether compounds 1 and 3 could additionally block RIPK3-dependent inflammation in neutrophil-associated diseases (Shi et al., 2015; Weinlich et al., 2017; Wang et al., 2018; Frank and Vince, 2019; Broz et al., 2020) remains unclear and requires additional experimentation.

We used a pharmacological approach to understand a non-apoptotic death pathway in human neutrophils. Such an approach has obvious limitations because pharmacological inhibitors can have pleiotropic effects. Compounds 1 and 3 might be able to block serine proteases present in azurophilic granules, which can also be involved, besides mitochondria, in death processes of neutrophils (Conus et al., 2008). However, we obtained no evidence that the compounds 1 and 3 would indeed target either of the granule serine proteases HNE, CatG or PR3 within human neutrophils. Therefore, the identification of the putative serine protease, which acts proximal to the RIPK3-MLKL complex in these cells, remains to be identified. In spite of this open question, our data suggest that serine proteases could represent potential therapeutic targets in neutrophil-associated disorders. Being able to prevent necroptotic-like neutrophil lysis, compounds 1 and 3 might be suitable candidates to treat such inflammatory diseases. Moreover, their chemical structures should provide additional hints for drug design and development.

## Data Availability Statement

The raw data supporting the conclusions of this article will be made available by the authors, without undue reservation, to any qualified researcher.

## Ethics Statement

The studies involving human participants were reviewed and approved by Ethics Committee of the Canton Bern. The patients/participants provided their written informed consent to participate in this study.

## Author Contributions

The study was designed by H-US and IM-R. The synthetic work and the chemical characterization of the compounds was conducted by DA and AO. The neutrophil work was conducted by XW. and SY. The manuscript was written by XW, IM-R, and H-US. All authors analyzed the data, read the manuscript, and gave approval to the final version.

## Funding

This work was supported by the Swiss National Science Foundation (31003A_173215 to SY. and 310030_184816 to H-US.), the European Union Horizon 2020 Research and Innovation Program (Marie Sklodowska-Curie grant No. 642295; MEL-PLEX), and by Slovenian Research Agency (P2-0208 to IM-R, AO, and DA).

## Conflict of Interest

The authors declare that the research was conducted in the absence of any commercial or financial relationships that could be construed as a potential conflict of interest.

## Abbreviations

CatG, cathepsin G; DPI, diphenylene iodonium; FRET, fluorescence resonance energy transfer; GaM, goat anti-mouse; GM-CSF, granulocyte/macrophage colony-stimulating factor; HNE, human neutrophil elastase; MAPK, mitogen-activated protein kinase; MLKL, mixed lineage kinase domain like pseudokinase; NADPH, nicotinamide adenine dinucleotide phosphate; PI, propidium iodide; PI3K, phosphatidylinositol 3′-kinase; PR3, proteinase 3; PS, phosphatidyl serine; RIPK, receptor-interacting protein kinase; ROS, reactive oxygen species; TNF, tumor necrosis factor; DAMPs, danger-associated molecular patterns; TPCK, N-*p*-Tosyl-l-phenylalanine chloromethyl ketone; TLCK, Nα-Tosyl-l-lysine chloromethyl ketone hydrochloride; TNF, tumor necrosis factor.
